# Feed resources, feeding system and feed balance of dairy cattle in Chencha District, Southern Ethiopia

**DOI:** 10.1002/vms3.70019

**Published:** 2024-09-26

**Authors:** Kechero Yisehak, Kore Adane

**Affiliations:** ^1^ Department of Animal Science Arba Minch University Arba Minch Ethiopia; ^2^ Livestock and Fishery Research Center Arba Minch University Arba Minch Ethiopia

**Keywords:** altitudes, dairy cattle, feed balance, feed resources, feeding system

## Abstract

**Background:**

Dairy cattle feed supplies in the tropics, and particularly in Ethiopia, are mostly derived from base diets (natural and improved grasslands, agricultural wastes, fodder trees and shrubs), agro‐industrial by‐products and non‐conventional feeds. The availability of major feed resource kinds, sources and dairy cattle feeding practises is primarily influenced by agro‐ecology and season, as well as their interactions.

**Objectives:**

The research was carried out in the Chencha District of Gamo Zone, Southern Ethiopia, with the goals of assessing the available, feeding system, opportunity, limits and copying strategy of dairy cattle production, as well as estimating feed balance.

**Methods:**

Data were gathered through primary and secondary data sources, field observations, key informant interviews, focus group talks and individual interviews. The survey data were stratified into altitudes, coded and analysed using the Statistical Package for Social Sciences version 20 and the general linear model approach. Cross‐tabulation was used to examine the statistical variance of categorical data.

**Results:**

The presence of different feed resource types, distinct agro‐ecologies (*p* < 0.05) and crop residue accessibility were the major opportunities for dairy cattle production in the study district, but land scarcity, a lack of dry season forages and land degradation due to erosion were the major constraints for dairy cattle feed production. In the research district, the main feed resource–copying approaches were reducing the number of dairy animals, saving optional feeds and purchasing optional feeds. The total mean of dairy cow feed supply to tropical livestock unit in terms of dry matter (DM) production each year was 3.86 t of DM/HH/annual, with a negative feed balance of 8.09 t that varied with seasons and agro‐ecology (*p *< 0.05).

**Conclusions:**

According to the findings of this study, the low production of dairy cattle in the district is clearly related to the scarcity of feed resources. To address these issues, alternative feed production technologies should be implemented, such as farmers practising forage development on their own crop land and collecting crop residues during crop harvesting times and storing them under shed; the nutritive value of different types of fodder trees and shrubs should be further determined in the future and feed storage methods, particularly hay and silage, should be used when there are an abundance of feed resources available.

## Graphical abstract

The main obstacles to dairy cattle production at all elevations and seasons, as determined by field measurement and the cross‐sectional questionnaire developed for this study, are soil degradation from erosion brought on by overstocking, land scarcity as a result of a high human population and a lack of forages, especially during the dry season. Of the feed supply gained from both on‐farms. In all, feed shortages of approximately 44.1%, 27.2% and 27.1% per year were noted in DM, ME and DCP, respectively.

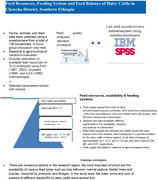

## 1 INTRODUCTION

Despite having the highest cattle population in Africa (Hunduma, 2012), Ethiopia's production is low. The low output is mostly the result of numerous limitations (Belay & Janssens, 2016; Getahun, 2012). Among these obstacles, a lack of sufficient quantity and quality feed ingredients has been cited as a major hindrance to the development of livestock production in Ethiopia (Alemayehu et al., 2017; Belay et al., 2011). Furthermore, Adugna et al. (2012), Alemayehu (2006), Yisehak et al. (2016) and Kechero and Geert (2014) showed that feed supply deficit was a key bottleneck for farmers in Ethiopian livestock production. Natural pasture and agricultural leftovers are the most important types of feed resources (Adugna et al., 2012).

In Ethiopia, crop wastes, such as teff, barley and wheat, account for 95% of animal feed (FAO [Food and Organization of United Nations], 2018). However, the contribution of feed resources is dependent on agro‐ecology, crop kinds produced, accessibility and production method (Ahmed et al., 2010; Seyoum et al., 2001; Thornton & Gerber, 2010). According to Zewdie (2010), most livestock production areas in the country have not yet addressed the assessment of the amount and quality of available feed supplies in relation to animal requirements. In Ethiopia, annual dry matter (DM) production could only meet two thirds of total DM requirements of livestock, resulting in animals losing condition during the dry season, which is an indicator of feed scarcity and suggests that livestock production and productivity are constrained by feed scarcity. In this regard, the annual requirements of the total tropical livestock unit (TLU) were exceeded by the availability of DM, crude protein (CP) and metabolizable energy (ME) from various feed resources (Kechero and Geert, 2014). As a result, energy and protein are the two most important limiting factors for livestock output (McDonald et al., 2010). According to a preliminary feed supply–demand assessment conducted in Ethiopia, the feed deficiency was 10% reported as DM, but 45% and 42% expressed as ME and CP, respectively (FAO, 2018). These data emphasise the importance of fodder conservation in order to ensure availability and quality throughout seasons and locations.

The CP level of pastures is lower than the value of forage CP content (7%), which would fulfil ruminant maintenance requirements (McDonald et al., 2010). The increasing need for agricultural land to provide food for humans in Ethiopia reduces the quantity of area available for natural grazing and forage production (Adugna et al., 2012; Alemayehu, 2005). As a result, the importance of natural pasture grazing land size is declining and shrinking (Yayneshet, 2010). In the case of Chencha District, as in other densely inhabited zones of Ethiopia, major feed resource production is dropping throughout the year. That is, of course, related to the scarcity of feed resources. As a result, assessing feed resources aids in the creation of effective intervention methods to increase feed quality, feed usage efficiency and dairy cattle productivity. As a result, this study was conducted to assess the opportunities, constraints and coping strategies of dairy cattle feed resources, feeding system and estimate the balance between total DM, CP and energy supply and nutrient requirements in Chencha District of Gamo Zone of Southern Ethiopia.

## 2 MATERIALS AND METHODS

### 2.1 About the study area

The study was conducted at Chencha District, which is located in Gamo Zone of Southern Ethiopia. The study was conducted at Chencha District, which is located in Gamo Zone of the Southern Nations, Nationalities and Peoples Regional State of Ethiopia. The district encompasses 50 *kebeles*, which has 45 rural *kebeles* and 5 peri‐urban *kebeles*. The altitudes of the district range from 1600 to 3250 m above sea level (masl). The district has two agro‐ecological zones, *woinadega* (1600%–2300% masl, 18%) and *dega* (2300%–3250% masl, 82%), whose total area covers an estimated area of 445 km^2^. The total population of the district was about 111,680, of which 51,307 were men and 60,373 were women, with an urban population of nearly 12% (Amene & Tesfaye, 2015). Based on the World Vision Ethiopia annual report (2015), the major means of livelihood for the district is mainly subsistence agriculture followed by traditional weaving and casual labour employment. In the study district, the population of cattle, horses, sheep, goats, donkeys and mules are 111,022, 7734, 198,159, 41,692, 328 and 864, respectively. The rainfall distribution in Chencha varies from year to year and across seasons. The annual rainfall distribution in the district varies between 900 and 1200 mm. The minimum temperature in the district ranges from 11 to 13°C, whereas the maximum temperature is in the range 18–23°C. The farming system in the district is a mixed farming system where the crop sub‐system and the livestock sub‐system are equally important to each other.

### 2.2 The study design

Primary and secondary data were gathered using qualitative and quantitative research approaches that included a structural questionnaire, field measurement, individual interviews and group discussions. Primary data on households, household herds, land holdings, key feed resources, opportunities, restrictions, dairy cattle feed resource copying tactics and feeding systems were obtained. Secondary data on climate, topography, agro‐ecology, human population and livestock population were gathered from the district office and other papers. The researcher facilitated the group talks at all sites, which included one group in each kebele. Multistage sampling strategies were used in this investigation. The purposively sampling technique was used in the first stage to choose districts based on dairy cattle potential. In the second stage, three kebeles from each district were chosen using a stratified sampling technique based on altitude range. In the third step, the head of kebeles picked a list of households in each kebeles using systematic random sampling procedures and estimated using the probability proportional to sample size methodology (Cochran, 1977):

$$no=\frac{Z^{2}\;\times \;P\;\times \;q}{d^{2}}$$

where no is desired sample size according to population greater than 10,000, *Z* = 1.96 for 95% confidence level, *P* = 0.1 for 10% proportion of the population, *q* = 1–0.1 and *d* = degree of accuracy desired at 5% error term. A total of 138 households (113 from highland and 25 from midland) from three kebeles were interviewed.

### 2.3 Livestock population

To estimate the feed balance of the study district, livestock population data were collected via questionnaire and secondary data sources (in the office of wereda) and converted to total TLU using (FAO, 1987, 2002a; Gryseels, 1988; ILCA, 1990) methodology. Total available DMs from natural pasture, crop residues, crop aftermath and fallow land, fodder tree and shrubs, enhanced forages and non‐convectional feed stuffs were compared to the yearly DM requirements of dairy cattle populations in the research area. During the study, data on the dairy cattle population were acquired from a questionnaire distributed to household heads. To compare the number of dairy cattle populations in the area with the DM requirements of the dairy cattle population based on the daily DM requirements for maintenance of 1 TLU (250 kg livestock) that consumes 2.5% of its body weight is 6.25 kg DM/day or 2281 kg DM per year and a CP content of 160 g/kg DM and 32.1 MJ ME/kg DM diet (MAFF, 1975; Jahnke, 1982).

### 2.4 Estimation the quantity of available feed resources

The quantity of feed resources in the research region was evaluated using information received from respondents during the survey on crop residues, grazing fields, fodder trees and bushes, enhanced forages and non‐convectional feed stuffs. The amount of crop leftovers used as livestock feed was calculated using standard conversion factors developed by various researchers. Wheat, barley and teff straw conversion factors are 1.5 per unit weight grain yield, whereas maize and haricot bean conversion factors are 2.0 and 1.2, respectively (Adugna, 1990; FAO, 1987). The DM output of grazing pasture was evaluated using the FAO (1987) multiplier factor of 2.0 t/ha. Crop aftermath grazing potential was assessed using a mean of 0.5 t/ha, whereas enhanced forages and fallow grazing land were 8 and 0.7 t/ha/year, respectively (Alemayehu, 2002; FAO, 1987). Based on respondents’ information, the quantity of additional non‐convectional feed stuffs with no obvious conversion factors can also increase the quantity of DM for cattle. The amount of theoretically accessible DM from enset (*Ensete ventricosum*) leaf and leaf midribs used for animal nutrition was approximated using a mean of 8 t/ha of enset‐growing area. The biomass production of shrubs and trees’ potential fodder yield was evaluated by measuring stem circumference with a measuring tape and using Petmak's (1983) equation. Accordingly, leaf yield of fodder trees was estimated by the allometric equation log *W* = 2.24 log trunk diameter (DT)‐1.50, where *W* is leaf yield in kilogrammes of dry weight and DT is trunk diameter (cm) at 130 cm height. Similarly, DT was obtained from DT = 0.636*C*, where the *C* = circumference in centimetres (cm). For the leaf yield of a shrub, the allometric equation is log *W* = 2.62 log DS‐2.46, where DS is stem diameter in cm at 30 cm height.

### 2.5 Opportunities, constraints and copying strategies of feed resources

For parameters required ranking, indices were calculated by using Microsoft office Excel 2007 to provide ranking of opportunities, constraints and coping strategies for the dairy cattle feed resources produced in the study district. Index was computed with the principle of weighted average according to the following formula as employed by Musa et al. (2006):

$$\begin{array}{ccc} \mathrm{Index} & = & \mathrm{Rn}\;\times \;C1+\mathrm{Rn}-1\;\times \;C2+\;\text{…}\;+R1\;\times \;\mathrm{Cn}/\sum \mathrm{Rn}\;\times \;C1\\ & & +\;\mathrm{Rn}-1\;\times \;C2\text{…}.R1\;\times \;\mathrm{Cn} \end{array}$$

where Rn is the value given for the least ranked level (e.g. if the least rank is fifth rank, then Rn = 5, Rn−1 = 4 and … R1 = 1). Cn is the count of the least ranked level (in the above example, the count of the 5th rank = Cn, and the counts of the 1st rank = C1).

### 2.6 Data analysis

All perceived and measurable data were analysed with SPSS version 20. The statistical variance of categorical data was examined using cross tabulation, assuming *p* < 0.05; however, descriptive statistics for numerical data were compared using the general linear model approach. To show the findings of perceptions and measurements, descriptive statistics, such as means, percentages and standard error of the mean, were utilized. The following statistical model is effective for evaluating dairy cattle feed resources, feeding systems and feed balance:

$$Y_{ijk}=\mu +\alpha_{i}+\beta_{j}+\alpha \beta_{ij}+\sum_{ijk}$$

where $\;Y_{ijk}$ is the total observation due to i and j; μ is the overall mean; $\alpha_{i}$ is the $i_{th}$ effect of agro‐ecology (altitude region, i = 1&2); $\beta_{j}$ is the $j_{th}$ effect of season (season, j = 1&2); $\alpha \beta_{ij}$ is the interaction of altitude and season and ${∼}_{ijk}$ is the random error.

## 3 RESULTS AND DISCUSSION

### 3.1 Household characteristics of respondents

In the current study district, the total mean family householding size was about 5.75 headed (Table 1). The average family size in both altitudes was significantly different (*p *< 0.05). In general, the average family size of the respondents in the study district was greater than the national average family size of rural areas (4.9) per household (CSA, 2011) and closely related to Emana et al. (2017), who reported an average family size of 5.37 in the Abol District of the Gambella region, Ethiopia. The educational status of respondents varied significantly (*p *< 0.05) among altitudes. As a result, the highest level of illiteracy was found in mid‐altitude (84%). These illiteracy levels in society pose problems to the modernization of dairy cattle feed production. According to group discussion information, the illiteracy levels of the society are the challenges on modernization of dairy cattle feed production, which requires continual training to enable livestock productivity to move forward. Yisehak et al. (2013) discovered a similar pattern in three districts in Jimma Zone, Southwest Ethiopia.

**TABLE 1:** Household characteristics of the respondents in the study district.

Characteristics	HIGHL	MIDL	Overall	*p‐*Value
Sex of the household,%				
Male	34.50	52	43.25	0.022
Female	65.50	48	56.75	0.022
Age the respondent years,%				
29–40	30.09^a^	28^b^	29.71	0.041
41–60	62.83^a^	44^b^	59.42	0.009
61–87	7.08	28	10.87	0.000
Mean family size				
Mean	5.46^b^	6.96^a^	5.75	0.033
Educational status, %				
Literate	37.20	16.00	33.30	0.005
Illiterate	62.80	84.00	66.70	0.005

Abbreviations: HL, highland; ML, midland; SEM, standard error of means.

^a,b^Means with different superscripts in the same row of altitude are significantly different (*p* < 0.05).

### 3.2 Livestock holding in TLU of sampled households

According to Table 2, the overall mean of livestock in sampled households in the current study was 7.699 TLU per family. The mean livestock in TLU was lower in the highlands (5.634) than in the midlands (9.764). The high mean value of cattle in the midland was attributed to relatively larger land resource holding sizes as well as the growing of a large number of livestock for social rather than production value. Out of this livestock, the aggregate mean of cattle per family in the research area was 3.577. The current result's overall mean was lower than a study result provided by Sisay (2006) in Debark (5.1 ± 0.35) TLU, Layarmachiho (5.6 ± 0.38). However, it was greater than Mergia et al. (2014) (3.3) in the Baresa watershed and Mengistu et al. (2016) (3.05 ± 0.15). The local cattle holding TLU per household in the highlands is significantly lower (*p *< 0.05) than in the midlands. This was due to farmers holding more local oxen for ploughing and threshing purposes in the midland than crossbreed oxen, so crossbreed oxen require more feed, whereas crossbreed cattle (LLU), particularly HF and jersey holdings per household in the highland were significantly higher (*p* < 0.05) than in the midland. Except for native oxen and calves, the influence of altitude showed a significant difference (*p* < 0.05) in the research region in terms of local and crossbreed cattle. Furthermore, the ease of access to required inputs, such as veterinary services and agro‐industrial by‐products, and the information gap among farmers were key factors.

**TABLE 2:** Livestock holding (in tropical livestock unit [TLU]) of sampled households.

	Altitude		Season		Mean	*p*‐Value		
Parameter	HL	ML	Ds	Ws	Overall	*α*	*β*	*α* × *β*
Cows								
Local	0.474^b^	0.704^a^	0.492	0.687	0.622	0.023	0.053	0.192
Cross	1.235^a^	0.432^b^	0.673	0.993	0.819	<0.000	0.099	0.561
Heifers								
Local	0.119^b^	0.242^a^	0.181	0.181	0.181	0.008	0.899	0.099
Cross	0.360^a^	0.048^b^	0.204	0.204	0.204	0.003	0.899	0.892
Bulls								
Local	0.277^b^	0.748^a^	0.625^a^	0.400^b^	0.193	0.002	0.004	0.006
Cross	0.748^a^	0.102^b^	0.210^a^	0.194^b^	0.388	0.004	0.004	0.006
Oxen								
Local	0.165	0.220	0.222	0.164	0.513	0.410	0.378	0.378
Cross	0.244^b^	0.532^a^	0.417	0.358	0.597	0.007	0.582	0.582
Calf								
Local	0.135	0.140	0.138	0.138	0.138	0.827	0.992	0.825
Cross	0.248^a^	0.096^b^	0.172	0.172	0.172	0.002	0.099	0.561
TLU of local cattle								
Local	1.237^b^	2.054^a^	1.657	1.634	1.922	0.003	0.880	0.023
TLU of cross cattle								
Cross	2.342^a^	1.512^b^	2.054	1.799	1.643	0.007	0.404	0.547
**Total cattle**								
**Both**	**3.579** ^a^	**3.566** ^b^	**3.712** ^a^	**3.433** ^b^	**3.577**	**0.003**	**0.040**	**0.003**
Sheep	0.931^b^	2.610^a^	0.215	0.212	0.214	<0.000	0.888	0.753
Goat	0.894^b^	1.914^a^	2.147	0.147	0.147	<0.000	0.986	0.576
Donkey	0.045^b^	1.453^a^	1.100	0.097	0.099	<0.000	0.928	0.692
Mule	0.031^b^	0.671^a^	0.100	0.103	0.101	<0.000	0.904	0.904
Horse	0.153^a^	0.131^b^	0.130	0.154	0.142	0.036	0.608	0.689
Poultry	0.023	0.041	0.026	0.011	0.032	0.012	0.036	0.621
**Sheep, goats, poultry and equine**	2.055^b^	6.198^a^	4.899	3.356	4.126	0.025	0.676	0.431
**Total livestock**	**5.634** ^b^	**9.764** ^a^	**6.789** ^a^	**8.610** ^b^	**7.699**	0.006	0.564	0.221

*Note*: Beta (greek letter) referes to the effects of seaon on dry matter supply of feed resources.

Abbreviations: αβ_ij_, the interaction of altitude and season; DS, dry season; HL, highland; ML, midland; WS, wet season, the (altitude region), the effect of season.

^a,b^Means with different superscripts in the same row of altitude (a, b) and also theseasons (a, b) separately are significantly different (*p *< 0.05).

### 3.3 Feeding system of dairy cattle in sampled households in study district

Based on respondents’ responses, the feeding system was significant (*p* < 0.05) across elevations in the study area; however, season had no effect (*p* > 0.05) on dairy cattle feeding system (Table 3). As a result, 46.9% of highland tether feeding systems and 48% of allow‐to‐graze feeding were more common in highland and midland altitudes, respectively. Indoor feeding, on the other hand, is less common in the highlands (11.5%), and no farmers were used in the midlands. This disparity was caused by differences in grazing field holding size among farmers. This was unconnected to research in Jeldu District, where 94.5%, 4.4% and 1.1% of respondents used the let‐graze, cut‐and‐carry and tether feeding systems, respectively (Bedasa, 2012).

**TABLE 3:** Feeding system of dairy cattle.

Feeding system in %	Altitudes		Season		Total		*p*‐Value	
	Highland (%)	Midland (%)	DS (%)	WS (%)	Altitude (%)	Season (%)	*α*	*β*
Grazing	6.19	48	43.5	7.20	54.19	13	0.002	0.904
Cut and carry	34.50	8	13	27.7	42.5	29.7	0.002	0.904
Tethering	46.9	44	33.3	56.9	90.9	47.1	0.001	0.904
Indoor feeding	11.50	–	9.40	8	11.5	9.7	0.001	0.904

Abbreviations: α*β_ij_, the interaction of altitude and season; DS, dry season; HL, highland; ML, midland; WS, wet season, the (altitude region), the effect of season.

### 3.4 Opportunity, constraint and copying strategy of feed resource scarcity

Table 4 shows the opportunity, restrictions and copying strategy of the principal dairy cattle feed resource. There are numerous options in the research region, the most important of which are the availability of various feed types, such as crop leftovers, natural pasture, fodder trees and bushes, industrial by‐products and forages. In the study area, the index score and rank of present of different feed stuffs for dairy cattle were ranked first. Following the presence of various feed stuffs, having appropriate and diverse agro‐ecology, and the presence of various forage varieties were ranked second and third, respectively. Despite the fact that there are numerous chances to improve dairy cattle feed resource production in the study region, there are a few obstacles that limit feed resource productivity. Respondents identified land scarcity due to high human population, a lack of dry season forages and land degradation due to erosion as the top three restrictions for dairy cattle production across altitudes and seasons. This is consistent with the findings of Azage et al. (2013) and Belay et al. (2011), who found that a lack of land was the most significant constraint on cattle production. However, based on replies from respondents in the study district, reducing livestock numbers, preserving optional feeds and acquiring optional feeds were the top three key copying methods of dairy cattle feed resources in the study district, placed first to third.

**TABLE 4:** Resource opportunities, limitations and copying tactics in connection to dairy feed.

	Altitudes				Season					
Parameters	Highland		Midland		Dry season		Wet season		Overall	
	Index	Rank	Index	Rank	Index	Rank	Index	Rank	Index	Rank
**Opportunity**										
Agro‐ecology	0.264	2	0.264	1	0.319	2	0.361	1	0.287	2
Presence of d/t forage varieties	0.208	3	0.333	2	0.416	1	0.305	3	0.253	3
Presence of d/t feed stuffs	0.528	1	0.403	3	0.263	3	0.333	2	0.458	1
**Constraints**										
Land scarcity	0.486	1	0.403	1	0.444	1	0.333	2	0.421	1
Shortage of dry season forages	0.278	2	0.319	2	0.291	2	0.458	1	0.328	2
Land degradation due to erosion	0.236	3	0.278	3	0.263	3	0.208	3	0.250	3
**Copying strategies**										
Minimize livestock number	0.444	1	0.444	1	0.416	1	0.388	1	0.347	1
Conserving optional feeds	0.333	2	0.361	2	0.361	2	0.333	2	0.287	2
Purchasing optional feeds	0.222	3	0.194	3	0.222	3	0.277	3	0.253	3

### 3.5 Production of different feed resources (in DM per year)

The average amount of DM (measured in tonnes) received from communal grazing land through the survey was 1.54 t of DM per year for highland and 1.16 t for midland, respectively, as shown in Table 5. DMs of 1.35 t were produced annually on communal grazing land in the research areas, on average. On the other hand, the mean annual production of DM from privately owned grazing land in the highlands and the midlands was 0.160 and 0.404 t, respectively. In the research areas, DMs of 0.282 t were produced annually from private grazing land on average. The results of the private grazing land in this study are less than those found in Sisay's (2006) report for the Lay Armachiho Woreda in the Gondar Zone, northern Ethiopia, which was 1.15 t/year.

**TABLE 5:** The dry matter (DM) supply of feed resources.

	Altitude		Season		Overall	*p*‐Value		
Feed supply, DM	High	Mid	Dry	Wet	Alt vs. Ses	*α*	*β*	*α* × *β*
Forest land	0.04^b^	0.185^a^	0.13	0.09	0.114 ± 0.015	<0.000	0.201	0.011
Crop aftermath	0.41^b^	0.483^a^	0.24^b^	0.38^a^	0.448 ± 0.013	0.007	<0.001	0.089
Communal	1.54	1.160	1.03	1.70	1.351 ± 0.078	0.783	0.070	0.985
Private land	0.16^b^	0.404^a^	0.32	0.24	0.283 ± 0.043	0.005	0.351	0.877
Fallow land	0.31	0.302	0.12	0.29	0.306 ± 0.050	0.935	0.101	0.359
Fodders	1.72^a^	1.017^b^	1.56	1.17	1.369 ± 0.108	0.001	0.073	0.012
Crop residue	1.01^b^	1.603^a^	1.76^a^	0.85^b^	1.31 ± 0.122	0.009	<0.00	0.851
Improved forage	0.112	0.078	0.212^a^	0.018^b^	0.183 ± 0.143	0.207	0.015	0.212
N‐conditional feeds	0.005	0.024	0.025	0.124	0.206 ± 0.150	0.021	0.173	0.067
Enset leaves	0.031	0.016	0.131^a^	0.026^b^	0.369 ± 0.208	0.001	<0.00	0.023
**Total DM supply**	**5.338**	**5.272**	**5.75**	**5.97**	**5.86** ± **0.321**	**0.908**	**0.050**	**0.581**

*Note*: Means with the different superscripts (a and b) in the same row of altitude and season are significantly different (*p *< 0.05). Beta (greek letter) referes to the effects of seaon on dry matter supply of feed resources.

In total, 1.307 t DM of crop wastes were produced annually by sample homes of various crop types. In the research area, the impact of altitude on agricultural residues was significant (*p* < 0.05). In mid‐altitude compared to high‐altitude, the mean yield of crop residue was higher. As a result, the crop residues in the different places vary because these agricultural residues produce differently at different altitudes. This outcome was consistent with the findings of Berihu et al. (2014), who noted crop residues in the Ganta‐Afeshum Woreda Eastern Zone of Tigray.

Livestock are permitted to graze the various crops’ stubble after harvest. As a result, at the study altitudes, a mean annual yield of 0.447 t of DM feed was derived from crop residue. The amount of stubble grazing land utilized in the current study was significantly affected by altitude change (*p* < 0.05). This resulted from variations in respondents’ landholding sizes. In the highland altitudes, these crop leftovers were primarily made from the leftover wheat, barley, bean and pea straw, whereas in mid‐altitudes, they were made from teff, wheat, maize and sorghum straw. The results of the current study were determined to be less significant than those of the Meta Robi District study (Endale, 2015).

By dividing the amount of available land by the conversion factors of 1.8 for fallow land grazing, the amounts of DM from fallow land were calculated (FAO, 1987). The effect of altitude change on the DM production of fallow grazing area was significant (*p*‐value 0.05). The mean DM yield of fallow grazing area at highland and midland was 0.312 and 0.302 t per year, respectively, according to data from household surveys.

According to FAO (1987), 0.7 is the conversion factor used to calculate the total DM production from forest area. As a result, an annual average of 0.113 t of feed DM was generated in the region. The altitude fluctuation has a substantial (*p*‐value 0.05) impact on the amount of DM produced by forests. The survey's findings show that at highland and midland, respectively, the mean DM output from forest land was 0.04 and 0.185 t. The Oromia regional state revealed that in the other study, conducted by Endale (2015) in the Meta Robi District, West Shewa Zone, the total DM output from forest land was 10.29, 2.62 t more than in the present study.

In the research region, the shrubs and trees used as fodder had the capacity to maintain animals all year round. The projected biomass yield of the trees and bushes in Table 5 averaged 1.369 t DM year. The mean estimated biomass yield of trees and bushes in the tested households was 1.72 t DM per year in the highlands and 1.017 t DM per year in the midlands, respectively.

Face‐to‐face interviews with respondents in the research areas revealed that non‐traditional feed sources were contributing to the dairy cattle feeds shown in Table 5. Although collecting actual data from families regarding their intake of non‐conventional feeds for their dairy cattle, it was not able to obtain a clear conversion factor for the current study. As a result, meeting the needs for feeding dairy cattle with non‐traditional foods such as enset stem, areke, tela and kitchen scraps.

### 3.6 Nutrient supply, requirements and estimated balance of dairy cattle

According to the daily DM requirements for maintenance of 1 TLU (250 kg livestock), which consumes 2.5% of its body weight, 6.25 kg DM/day and a CP content of 58 g/kg DM and 5.2 MJ ME/kg DM diet is used (Kearl, 1982), whereas 1 LSU (450 kg livestock), which consumes 10 kg DM/day/animal and a CP content of 70 g/kg DM and 8.368 MJ ME/kg DM diet for maintenance, is used (Winrock International, 1992). So, in the current study, DM, CP and ME availability, requirements and estimated balance of feeds for dairy cattle are presented in Table 6. The mean DM supply of feed from natural pasture grazing, crop residues, crop aftermath grazing, fallow land, woodland grazing, non‐conventional feeds, fodder trees and shrubs was 4.17 and 3.55 t/year in highland and midland, respectively. The overall mean annual DM requirement of livestock and dairy cattle was 11.96 and 8.76 t, respectively. But the overall mean of annual DM supply of livestock and dairy cattle feed was 3.86 t. The overall mean DM of feed supply in the study districts for livestock as well as was below the requirements. So, the requirements of livestock as well as dairy cattle feed were not balanced with the yield from different feed resources in study area. The effect of altitudes was significant (*p *< 0.05) on the mean of DM, ME and CP requirements and feed balance in the study altitudes. However, it was not significant (*p *> 0.05) on the mean of DM, ME and CP supply of livestock and dairy cattle. In agreement with current study, Bedasa (2012) also indicated that the annual DM production was below the annual livestock requirements of the Blue Nile basin in Ethiopia.

**TABLE 6:** Nutrient availability, requirements and estimated balance of feeds for livestock.

	Dairy cattle							
	Altitudes		Seasons		Overall	*p*‐Value		
Nutrients	Highland	Midland	Dry	Wet	MSE	*α*	*β*	*α* × *β*
DM supply, tons	4.17^a^	3.55^b^	3.75^b^	3.97^a^	3.86	0.038	0.048	0.551
DM required, tons	7.79^b^	9.73^a^	9.41	8.11	8.76	0.007	0.071	0.002
**DM balance, tons**	**−3.6^a^**	**−6.18^b^**	**−5.65**	**−4.14**	**−4.89**	**0.002**	**0.060**	**0.011**
DP supply, tons	0.66^a^	0.56^b^	0.59	0.63	0.614	0.038	0.481	0.551
DP required, tons	2.24	2.29	2.26	2.27	2.26	0.812	0.941	0.037
**DP balance, tons**	**−1.6^a^**	**−1.73^b^**	**−1.65**	**−1.64**	**−1.65**	**0.047**	**0.944**	**0.030**
ME supply, tons	79.7^a^	67.8^b^	71.74	75.78	73.76	0.038	0.481	0.551
ME required, tons	86.43	97.32	270.9	273.1	272.01	0.812	0.941	0.037
**ME balance, tons**	**−6.73^b^**	**−18.01^a^**	**−253^b^**	**−115^a^**	**−183.5**	**0.004**	**0.002**	**0.001**

	Livestock							
	Altitudes		Seasons		Overall	*p*‐Value		
Nutrients	Highland	Midland	Dry	Wet	MSE	*α*	*β*	*α* × *β*
DM supply, tons	4.17^a^	3.55^b^	3.75^a^	3.97^b^	3.86	0.038	0.048	0.055
DM required, tons	23.064	16.035	23.624	15.48	19.55	0.173	0.115	0.691
**DM balance, tons**	**−18.89**	**−12.48**	**−19.87**	**−11.51**	**−15.69**	**0.095**	**0.096**	**0.906**
DP supply, tons	0.66^a^	0.56^b^	0.59	0.63	0.614	0.038	0.481	0.551
DP required, tons	0.997	1.092	1.181^a^	0.908^b^	1.044	0.254	0.001	0.012
**DP balance, tons**	**−0.337**	**−0.532**	**−0.62^a^**	**−0.28^b^**	**−0.43**	**0.254**	**0.001**	**0.119**
ME supply, tons	79.7^a^	67.8^b^	71.74	75.78	73.76	0.038	0.481	0.551
ME required, tons	135.47^a^	98.402^b^	141.89	91.98	116.939	0.373	0.230	0.438
**ME balance, tons**	**−55.77^a^**	**−30.60^b^**	**−70.15^a^**	**−16.2^b^**	**−43.18**	**0.006**	**0.021**	**0.056**

*Note*: Means with the different superscripts (a and b) in the same row of altitude (a and b) and season (a and b) are significantly different. Beta (greek letter) referes to the effects of seaon on dry matter supply of feed resources.

Abbreviations: DM, dry matter; ME, metabolizable energy.

## 4 CONCLUSION AND RECOMMENDATION

In the research region, the fodder trees and shrubs had the potential to maintain dairy cattle throughout the year, particularly during the dry season. The projected biomass yield of trees and shrubs in the highland area was 1.737 t DM/HH/year on average. *Vernonia amygdalina*, *Ficus sur*, *Dombeya torrida*, *Maesa lanceolata* and *Ficus vasta* were among the many rich feed sources for dairy cattle in the midland parts of Chencha District, particularly during the dry season. Negative balance was found in both study altitudes because the overall mean of DM, ME and CP requirements for livestock and dairy cattle was higher than the actual feed produced. It took 11.96 and 8.76 t of DM feed per year, respectively, to maintain the tested households’ overall mean livestock and dairy cattle populations of 7.699 and 3.573 TLU, respectively. Only 44.06% of the yearly maintenance DM needs of dairy cattle units per household can be met by the current feed supply. As a result, in order to address the issue of feed shortages in the research area, the respondents engaged in destocking, the conservation of agricultural leftovers and the purchase of different feed types from nearby farmers. The research area's ideal livestock and dairy cattle cannot be satisfied by the current feed supply. Because of this, efforts for enhancing feed quality as well as the promotion of local and enhanced forage production should be implemented. Additionally, more research and development efforts should be planned to advance the local forage varieties, both those that are native and those that have been enhanced. Due to land scarcity, ignorance, a lack of forage seeds and lack of interest, the current study found that better forage for livestock and dairy cattle feed had a minimal impact. To solve this issue, nurseries should be constructed in potential kebeles of the districts, where they can share information with farmers and distribute these feeds. Although the nutritive value of such feeds is unknown in the area, further research on their quality should be done in the near future. The area has promise for fodder plants and shrubs for their dairy cattle feeding.

## AUTHOR CONTRIBUTIONS

Kechero Yisehak conceptualized the idea of the study, curated the data, performed formal analysis and investigation, designed methodology, administered the project and wrote the original draft. Kore Adane acquired funding, performed writing, paper drafting, reviewed and edited the manuscript.

## CONFLICT OF INTEREST STATEMENT

The authors declare no conflicts of interest.

### ETHICS STATEMENT

The authors affirm that the journal's ethical rules, as stated on the author guidelines page, were followed. However, the study did not use animals, but rather a questionnaire survey, field observation and measurement. As a result, data collection was carried out following a verbal agreement with the agricultural and locality administration offices, as well as the research's target farmers.

### PEER REVIEW

The peer review history for this article is available at https://publons.com/publon/10.1002/vms3.70019.

## Data Availability

The data associated with this research are available at https://zenodo.org/record/8296571 and can be accessed under the condition (License) of Creative Commons Attribution 4.0 International.
